# Supplementary material for: Feasibility test of per-flight contrail avoidance in commercial aviation
Source: Commun Eng. 2024 Dec 20;3:184. doi: 10.1038/s44172-024-00329-7 (PMC11659620; doi:10.1038/s44172-024-00329-7)
Supplement: Supplementary file 2 — Supplementary Information [file 44172_2024_329_MOESM2_ESM.pdf]

Supplementary Information for Feasibility Test of Per-Flight Contrail Avoidance in Commercial Aviation

Supplementary Table 1: Experiment results table

Trial Flight ID	Pair ID	AA Flight #	Date	Group Flag	Contrail formation	Origin Airport	Turnaround city
id-1279442204980825327	1013	AAL2114	4/7/2023	Treatment	0	DFW	SNA
id-1630557003953485365	1019	AAL359/AA1272	4/27/2023	Treatment	0	DFW	BZE
id-206278396954589293	1026	1048/2367	5/21/2023	Control	0	DFW	PNS
id-2240567770464453953	1029	2645/2932	5/26/2023	Control	1	PHX	SJD
id-245039482236718990	1034	2279	6/4/2023	Control	0	PHX	PVR
id-3021732248880410269	1000	982	1/16/2023	Control	0	DFW	ATL
id-3419098391407802672	1018	AAL0554	4/21/2023	Treatment	0	PHX	PVR
id-3522864736414155179	1001	353	1/16/2023	Control	0	DFW	PVR
id-3617378476420843301	1030	1271	5/29/2023	Treatment	0	DFW	TPA
id-3637362072336995394	1007	189/1683	3/10/2023	Control	1	DFW	MCO
id-4188975314109540893	1029	2645/2932	5/26/2023	Treatment	0	PHX	SJD
id-4251931904675004504	1004	2679	2/24/2023	Treatment	1	DFW	SRQ
id-4266751200797482767	1014	AAL1709	4/7/2023	Treatment	0	DFW	MCO
id-5156840351937990038	1010	535/2841	4/2/2023	Treatment	0	PHX	SJD
id-5578262632707376636	1017	AAL2496	4/21/2023	Treatment	1	DFW	PDX
id-5871402798939296391	1009	2932	3/26/2023	Treatment	0	PHX	SJD
id-6472907038231011042	1000	982	1/16/2023	Treatment	0	DFW	ATL
id-6524388267057616160	1018	AAL0554	4/21/2023	Control	0	PHX	PVR
id-7134667873761741507	1012	AAL2888	4/7/2023	Control	1	PHX	SNA
id-7349341453851072874	1030	1271	5/29/2023	Control	1	DFW	TPA
id-7372297730076407466	1016	AAL1156	4/16/2023	Control	1	PHX	EUG
id-8126931667611158490	1008	2112/433	3/17/2023	Treatment	0	LAX	DFW
id-8994676185677859117	1023	AAL554	5/6/2023	Treatment	0	PHX	PVR
id-9174139119764019977	1008	2112/433	3/17/2023	Control	0	LAX	DFW
id2034414633364111269	1025	2451/926	5/19/2023	Control	0	DFW	BZE
id2056219067563816106	1019	AAL359/AA1272	4/27/2023	Control	1	DFW	BZE
id2542245579920360986	1010	535/2841	4/2/2023	Control	1	PHX	SJD
id3131880188513765811	1025	2451/926	5/19/2023	Treatment	0	DFW	BZE
id329797977336080811	1023	AAL554	5/6/2023	Control	1	PHX	PVR
id3754694128305243252	1014	AAL1709	4/7/2023	Control	1	DFW	MCO
id3804382984315982628	1013	AAL2114	4/7/2023	Control	0	DFW	SNA
id3830403107545807200	1034	2279	6/4/2023	Treatment	0	PHX	PVR
id414870142547593523	1004	2679	2/24/2023	Control	0	DFW	SRQ
id5005706939919721775	1002	1273	2/17/2023	Treatment	1	DFW	RSW
id5172335236488176527	1026	1048/2367	5/21/2023	Treatment	0	DFW	PNS
id5615126350052254763	1016	AAL1156	4/16/2023	Treatment	1	PHX	EUG
id5761699602696592119	1027	2576	5/22/2023	Treatment	0	DFW	CZM
id6406562754134259949	1012	AAL2888	4/7/2023	Treatment	0	PHX	SNA
id7136926184930758565	1002	1273	2/17/2023	Control	1	DFW	RSW
id7390083454776565459	1001	353	1/16/2023	Treatment	0	DFW	PVR
id8256452031856397448	1017	AAL2496	4/21/2023	Control	0	DFW	PDX
id9013955756842448189	1027	2576	5/22/2023	Control	0	DFW	CZM
id907671101067364742	1009	2932	3/26/2023	Control	1	PHX	SJD
id972962394493208273	1007	189/1683	3/10/2023	Treatment	0	DFW	MCO

This table is available in csv format in the study's [Google Cloud bucket].

Supplementary Note 1: Links to visualizations

All html files used for labeling are publicly available in Google's visualizations repository.
(Log in with a gmail account, or another email registered with Google).
Files are available in other formats upon reasonable request.

Supplementary Note 2: Link to trial analysis

- The code to run the crossover trial analysis & reproduce our results can be found in our crossover_trial_analysis colab notebook.
